# Diversified diazotrophs associated with the rhizosphere of Western Indian Himalayan native red kidney beans (*Phaseolus vulgaris* L.)

**DOI:** 10.1007/s13205-014-0238-5

**Published:** 2014-07-24

**Authors:** Deep Chandra Suyal, Amit Yadav, Yogesh Shouche, Reeta Goel

**Affiliations:** 1 Department of Microbiology, College of Basic Sciences and Humanities, G.B. Pant University of Agriculture and Technology, Pantnagar, 263145 Uttarakhand India; 2 Microbial Culture Collection, National Centre for Cell Science, Pune University Campus, Ganeshkhind, 411 007 Pune India

**Keywords:** Red kidney beans, Himalayan agro-ecosystems, Rhizosphere, *nif*H, Bacterial diazotrophic ecology

## Abstract

Red kidney beans (RKBs) are one of the major components in the human diet of Western Indian Himalaya (WIH). Their cultivation in these habitats is strongly influenced by various biotic and abiotic stresses and therefore, there must be a selection of RKB associated microorganisms that are adapted to these harsh conditions. Seven cold adaptive diazotrophs from the same rhizosphere were isolated in our previous study to reveal the low-temperature associated proteins and mechanisms. However, the diversity and phylogenetic affiliations of these rhizosphere diazotrophs are still unknown. In this study, RKB rhizospheric soil from two different agro-ecosystems of WIH namely S1 (Chhiplakot, 30.70°N/80.30°E) and S2 (Munsyari, 30.60°N/80.20°E) were explored for the assessment of nitrogenase reductase gene (*nif*H) diversity by plating respective clone libraries SN1 and SN2. The RKB rhizosphere diazotroph assemblage was very diverse and apparently consists mainly of the genera *Rhizobium*, followed by unknown diazotrophic microorganisms. Deduced amino acid sequence analysis revealed the presence of diverse *nif*H sequences, affiliated with a wide range of taxa, encompassing members of the Proteobacteria, Actinobacteria and Firmicutes. Members of cyanobacteria, methanotrophs and archaea were also detected. To the best of our knowledge, this is the first major metagenomic effort that revealed the presence of diverse nitrogen-fixing microbial assemblages in indigenous RKB rhizospheric soil which can further be explored for improved crop yield/productivity.

**Electronic supplementary material:**

The online version of this article (doi:10.1007/s13205-014-0238-5) contains supplementary material, which is available to authorized users.

## Introduction

Agriculture is an important livelihood activity in the Himalayan high-altitude agro-ecosystems; preferring the natural farming over chemical based farming, therefore, causative for emergence of hilly agri lands as a gold mine for adaptable potential microorganisms. In these habitats, microbial nitrogen fixation is of particular interest since the low concentration of bio-available nitrogen is one of the key limitations for growth of plants and soil microorganisms (Duc et al. 2009). Our previous studies highlighted the prevalence of many important genes/proteins from icy heights of the Himalaya (Prema latha et al. 2009). Soni and Goel (2010) identified several *nif*H homologs from Western Indian Himalayan soil metagenomics and enlighten the *nif*H phylogeny. Recently, seven cold adapted bacterial diazotrophs from the RKB rhizosphere of WIH were isolated and proteome of the psychrophilic nitrogen-fixing *Pseudomonas migulae* strain S10724 for low-temperature diazotrophy was documented (Suyal et al. 2014). Here, same rhizosphere metagenome was analyzed to examine the ecological and biological patterns of Himalayan diazotrophs.

In WIH, RKB is home-grown high-protein food crop and is an integral part of the cuisine, *Rajmah*. Among all legumes, it constitutes a high amount of proteins (20–15 %), complex carbohydrates (50–60 %) and a better source for vitamins, minerals and poly unsaturated free fatty acids (Reddy et al. 2013). However, lower production and escalating prices of RKB may result in protein malnutrition, especially among those living below the poverty line. Being an organic state, use of chemical fertilizers for crop production is not recommended. Moreover, available plant growth promoting (PGP) bio-formulations may not be effective unless or otherwise supported by biotic and abiotic factors. Therefore, it has become imperative to unravel the native microbial community structure–function for sustainable agriculture plans.

Multitrophic interactions in the rhizosphere are always determined by plant-microbial interactions including both symbiotic as well as free living microorganisms (Philippot et al. 2013). The diversity of rhizobia nodulating RKB has been studied widely (Valverde et al. 2006; Diaz-Alcántara et al. 2013), but, scarce information is available about its rhizospheric diazotrophic diversity, especially from the Himalayan ranges. In this perspective, various agro-climatic zones of WIH were screened for assessing the diversity and community structure of the diazotrophs using *nif*H as a biomarker. *nif*H is the oldest existing functional gene in the history of evolution and its sequence is highly conserved among diverse microorganisms (Raymond et al. 2004). Furthermore, bacterial phylogeny based on sequence divergences of *nif*H are generally in agreement with the phylogeny inferred from 16S rRNA gene sequences, and, therefore, provides a practical means of classifying uncultivated diazotrophic microorganisms. The database for nitrogenase genes (specifically the *nif*H gene) has become one of the largest non-ribosomal gene datasets on uncultivated microorganisms (Zehr et al. 2003).

The primary objective of this study was to examine the ecological and biological patterns of N_2_-fixing microorganisms in a Himalayan RKB rhizosphere and utilize this information to provide the backbone for future studies. Moreover, *nif*H sequence clustering patterns will be beneficial in refinement of *nif*H phylogeny by enriching the respective database.

## Materials and methods

### Sampling sites and sample collection

**Fig. 1:**
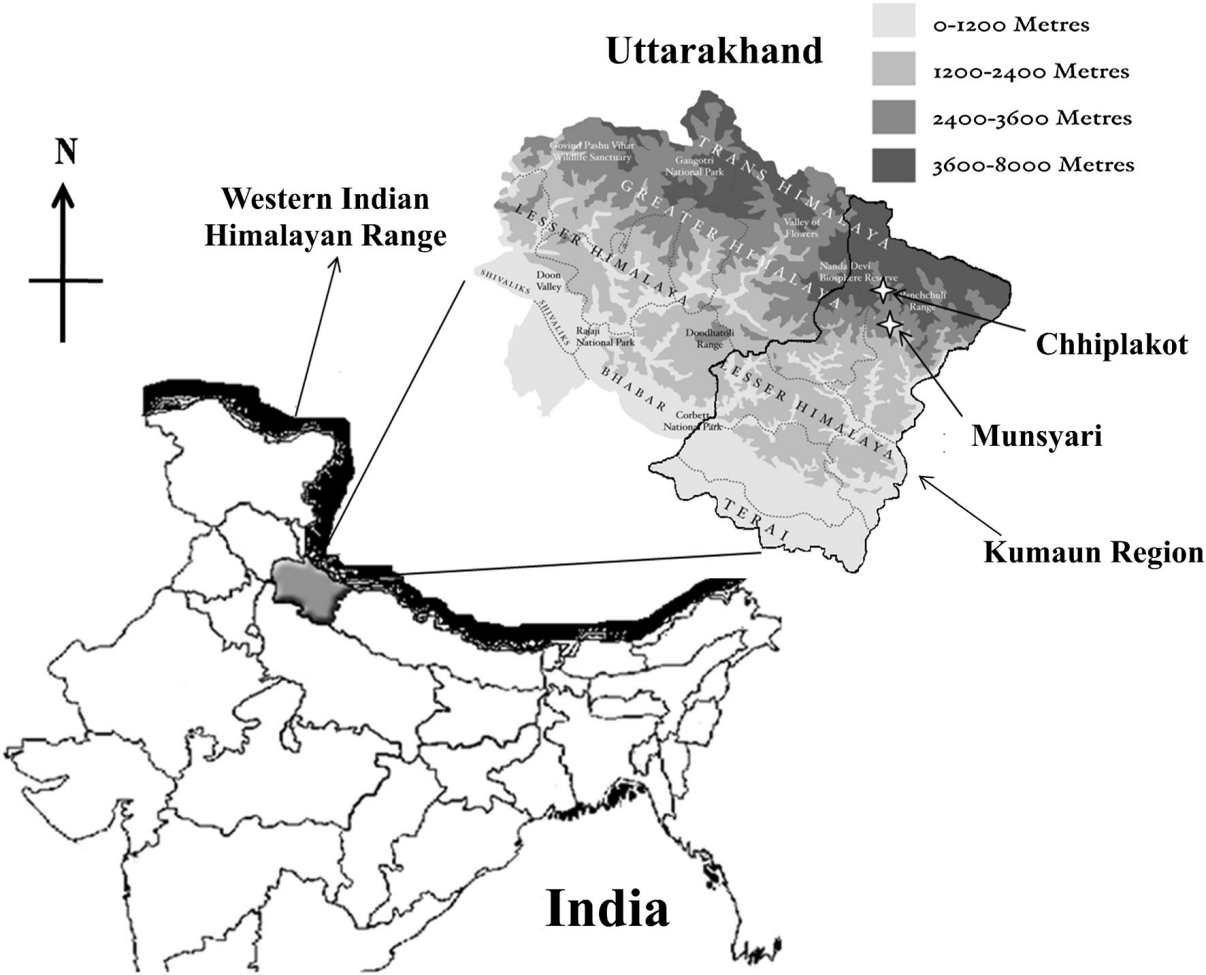
Geographical location of the sampling sites under study. *White colored stars* highlight the sampling locations Chhiplakot (S1) and Munsyari (S2) of Kumaun region of Uttarakhand, India. *Lighter* to *dark shaded area* indicates increasing altitude level from Tarai area to Trans-Himalaya

The sampling sites were located on the upper reaches of Kumaun Himalaya (Fig. 1). Rhizospheric soil samples (not deeper than 15 cm) were collected from the rhizosphere of Red Kidney beans (*P. vulgaris* L.) using sterile spatula in sterile polythene bags and transported to laboratory under sterile and cold conditions. Each soil sample was collected in triplicates which were later mixed to make a single composed sample per site. Samples for chemical analysis were stored at 4 °C, and samples for clone library analysis were stored at −20 °C till further use.

### Chemical analysis of the soils

Soil samples (1 g) were analyzed chemically at Accurate Analytical Laboratory, Pune (ISO 9001-2000; Certified by TÜV, Germany) for pH, total carbon (TC), nitrogen (TN), phosphorus (TP) and potassium (K) contents.

### Total soil DNA extraction

Total soil DNA was extracted from each 0.5 g (fresh weight) soil sample by using the Powersoil™ DNA isolation kit (Mobio Lab. Inc., USA), according to the manufacturer’s instructions. After extraction, DNA samples were quantified spectrophotometrically at 260 nm and used immediately for *nif*H library construction; remaining DNA was stored in TE buffer (10 mMTris, 1 mM EDTA, pH 8.0) at −80 °C till further use.

### Real-time PCR (qPCR) analysis

Copy number of *nif*H genes from collected soil samples was quantified using iCycleriQ™ Multicolor (Bio-Rad Lab, Hercules, USA) real-time polymerase chain reaction (qPCR) machine as described previously (Soni and Goel 2010). In brief, plasmid of the bacterium *Bradyrhizobium japonicum* USDA6 was isolated, quantified and used as a standard for qPCR analysis in serial dilutions (0.1–100 ng). PCR reactions was carried out in a volume of 25 μl containing 1X iQTM SYBR Green Supermix (Bio-Rad), 100 pmol of each *nif*H primer PolF (5′ TG GAY CCS AAR GCB GAC TC 3′), PolR (5′ ATS GCC ATC ATY TCR CCG GA 3′) and 50 ng of template DNA. Cycling conditions included 5 min of the denaturation step at 95 °C, followed by 30 cycles of 30 s at 95 °C, 30 s at 50 °C and 30 s at 72 °C (Poly et al. 2001).

### *nif*H library construction and sequence analysis

*nif*H genes were amplified as described earlier (Poly et al. 2001). The amplified 360-bp bands were excised from 1.2 % (w/v) agarose gel and purified using the Hipura™ Quick gel purification kit (Himedia). The polymerase chain reaction (PCR) product were analyzed on 1.2 % agarose gel and quantified spectrophotometrically at 260 nm. Purified PCR products were cloned into the pCR4-TOPO^®^ *Escherichia coli* vector (Invitrogen) by using the ‘TOPO TA cloning^®^’ kit (Invitrogen) for sequencing with ‘One Shot TOP10 Electro-Competent cells^®^’, according to the manufacturer’s instructions (Invitrogen) on the same day. Randomly selected colonies were picked and subjected to colony PCR in 25 μl of PCR mixture as described earlier (Poly et al. 2001). PCR products were PEG purified and sequenced in both directions using the AB dye terminator (Applied Biosystems) as described previously (Surakasi et al. 2010).

### Statistical and phylogenetic analysis of the *nif*H clone sequences

Phylogenetic analysis of the clone sequences was carried out as reported by Surakasi et al. (2010). All sequences were assigned to an operational taxonomic unit using distance-based OTU and richness (DOTUR) at 5 % sequence distance cutoff. The diversity of operational taxonomic units (OTUs) was further examined using rarefaction analysis. Coverage of *nif*H gene clone libraries and various other diversity indices were determined with paleontological statistics (PAST) version 1.77 (Cetecioglu et al. 2009). Shannon–Wiener Diversity Index (Zhu 2002) was used to calculate Shannon index (H0), evenness and the Simpson’s index (D). The sequences were then used to construct the phylogenetic tree using molecular evolutionary genetics analysis (MEGA) 5 (Tamura et al. 2011) in accordance with the interior test of phylogeny using a neighbor-joining algorithm.

### Nucleotide sequence accession numbers

The *nif*H clone libraries sequences reported in this paper have been deposited in the GenBank database under accession numbers JX154682 to JX154877.

## Results

### Physicochemical characteristics of the soil samples and *nif*H abundance

Two soil samples S1 and S2 were selected among 20 different RKB rhizospheric samples collected from various agro-climatic zones of WIH, on the basis of *nif*H abundance using real-time PCR (qPCR) technique (Table SM1). Bacterial gene abundances act as the indicators of various processes occurring in the respected ecosystem and therefore, real-time PCR becomes an effective and sensitive presumptive screening tool for estimating microbial abundance (Morales et al. 2010). The collection sites of S1 and S2 were from Chhiplakot, a place having lush stretches of velvety grass “*bugyals*” (alpine meadows) and Munsyari, located at the bottom of “Milam” glacier, respectively. Physicochemical analysis of S1 and S2 revealed the significant difference between the rhizospheric soils of both the agro-ecosystems. Large difference among the total nitrogen (N) and total organic carbon (TOC) content of both the soils were recorded. However, total phosphorus (P) and potassium (K) content differ less significantly (Table 1). The higher TOC content of S1 might be due to the higher biological activity in S1 in comparison to S2 as also supported by the higher counts of diazotrophs in earlier soil (Table SM1). Further, mountainous cold-temperate areas have high TOC content but large spatial variability, due to variable climate and vegetation (Li et al. 2010). Various studies have reported the influence of topography (Yoo et al. 2006), climatic conditions (Davidson and Janssens 2006), soil composition (Jobbágy and Jackson 2000), litter quality and its decomposition rate (Yang et al. 2005) and species composition or vegetation type (Schulp et al. 2008) on the spatial distribution of TOC.

**Table 1:** Physicochemical characteristics of the soil samples

Soil parameters	S1 (Chhiplakot)	S2 (Munsyari)
Altitude (m)	3,090	2,200
Soil texture	Fine, black	Fine, dark brown
pH	7.0	6.8
Total Kjeldhal nitrogen (TKN) as N (%)	0.4822	0.0512
Total phosphorus (P) as PO4 (%)	12.1020	13.1458
Potassium as K (%)	0.1701	0.2175
Total organic carbon (TOC) (%)	5.9035	1.6994
*nif*H clone libraries	SN1	SN2

### Cloning and analysis of *nif*H sequences

**Fig. 2:**
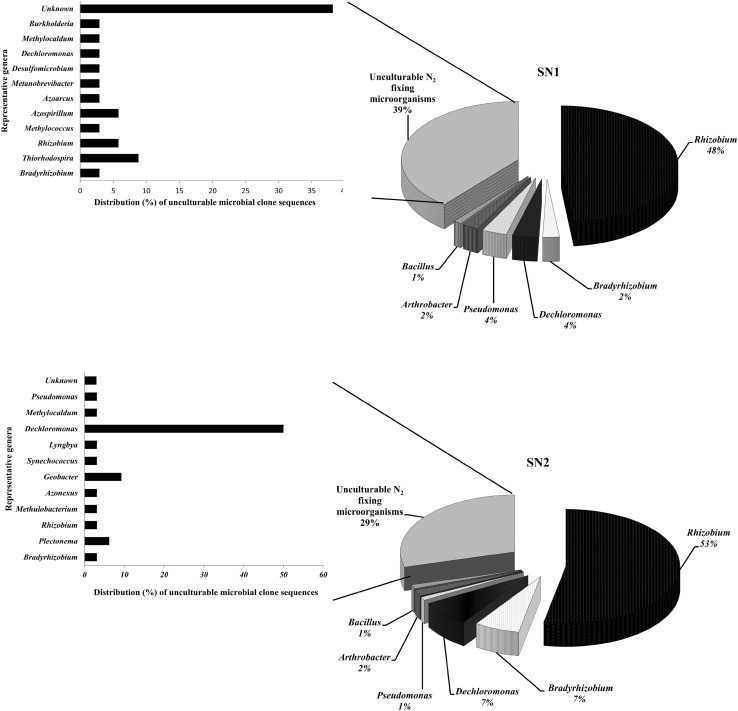
Diazotrophic bacterial community composition of the *nif*H libraries SN1 and SN2 constructed from the soils S1 and S2 collected from altitude of 3,090 and 2,200 m, respectively. Nearest cultivable representatives of the unculturable N_2_ fixing microbial clone sequences based on NCBI blast homology searching excluding uncultured/environmental sample sequences are also shown by *horizontal bar charts*. Values represent the percent distribution of the bacterial groups in the *nif*H libraries

A total of 196 positive clones were obtained from two clone libraries SN1 (87 sequences) and SN2 (109 sequences); constructed using the soils S1 and S2, respectively. A good fraction of the clone sequences belonged to unculturable N_2_ fixers (39 % in SN1 and 29 % in SN2). Upon excluding the uncultured/environmental sample sequences from the queries, most of them showed sequence homology with their nearest cultivable representatives (Fig 2). Thus, overall distribution of the clones indicated that Alphaproteobacteria were most predominant (>50 %) in both the libraries represented by genera *Rhizobium*, *Bradyrhizobium* and *Azospirillum* in SN1 and by *Rhizobium*, *Bradyrhizobium* and *Methylobacterium* in SN2. *Betaproteobacteria* were represented by the genera *Dechloromons*, *Burkholderia* and *Azoarcus* in SN1 while by *Dechloromons* and *Azonexus* in SN2. In case of Gammaproteobacteria, SN1 was found to be more diverse than SN2 by possessing genus *Pseudomonas*, *Thiorhodospira*, *Methylococcus* and *Methylocaldum.* Deltaproteobacterial group was represented by a lone genus *Desulfomicrobium* and *Geobacter* in SN1 and SN2, respectively. Furthermore, both the libraries possess single but similar members of Actinobacteria (*Arthrobacte*r) and Firmicutes (*Bacillus*). In addition to this, an archaeal *Methanobrevibacter* *nif*H sequence was also encountered in SN1. Cyanobacterial *nif*H sequences belonging to the genera *Lyngbya*, *Synechococcus* and *Plectonema* were exclusively observed in SN2 library.

### Statistical and phylogenetic analysis

**Fig. 3:**
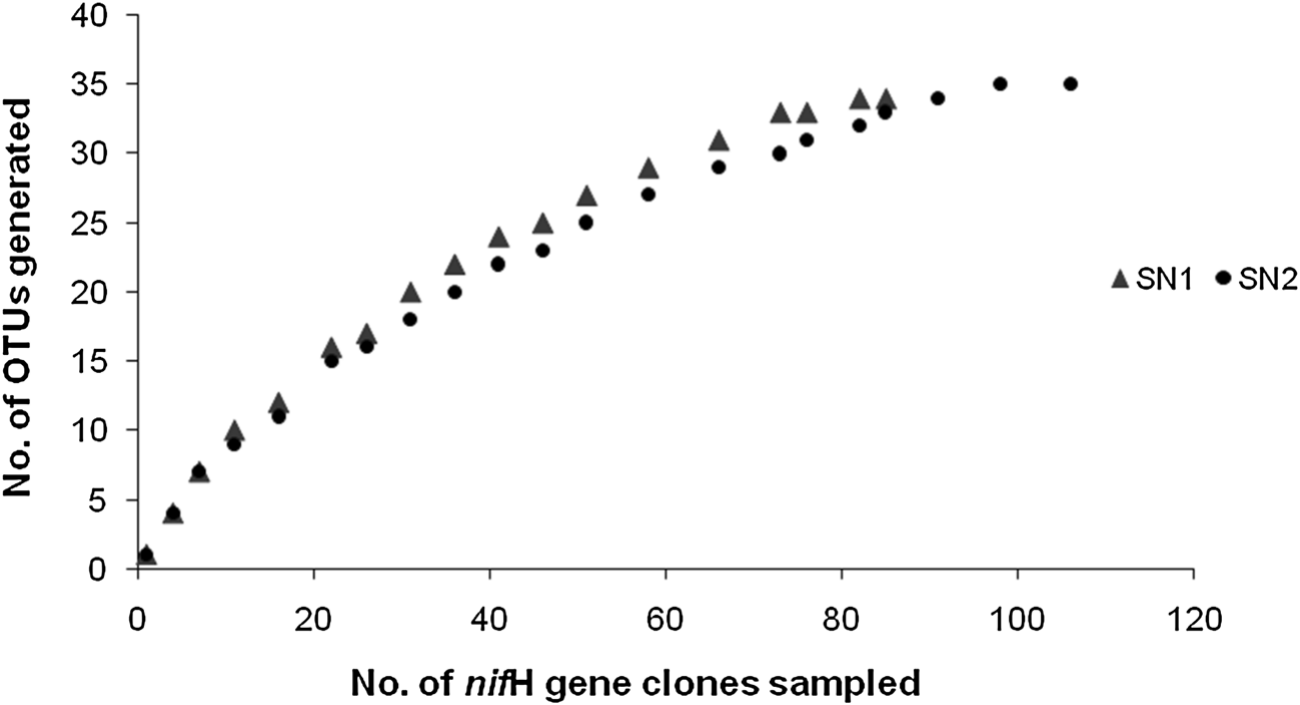
Rarefaction curves indicating the early saturation of observed OTUs in the libraries SN1 and SN2, respectively

Determination of operational taxonomic unit (OTU) is one of the preferred methods that are currently available for comparing diversity from different clone libraries. Diversity is a function of the number of OTUs present (OTU richness) and the evenness with which clones are distributed among these OTUs (OTU evenness) (Hurlbert 1971). Based on similarity criteria of ≥95 % clone libraries, SN1 and SN2 were categorized into 34 and 35 OTUs. The rarefaction curves in both the libraries were plateaued, indicating their saturation (Fig. 3). Both the libraries had similar richness and diversity with slight differences (Table 2). Shannon index indicated that the SN2 library (1.239) was more diverse than the SN1 (1.176). This index is increased either by having additional unique species, or by having greater species evenness (Shannon 1948). These results are well supported by the values of Margalef and Menhinick indices (higher in SN2) which estimate the species richness independently of the sample size (Magurran 2004). Moreover, the value of Berger–Parker Index was also higher in SN2. This Index expresses the proportional importance of the most abundant species (Berger and Parker 1970). Increase in the value of this index accompanies an increase in diversity and a reduction in dominance (Magurran 2004). For the Simpson index, values near zero corresponding to highly diverse or heterogeneous ecosystems and values near one corresponding to more homogeneous ecosystems (Simpson 1949) indicating the saturation risk of Western Himalayan diazotrophic diversity as the observed values of this index were 0.62 (SN1) and 0.61 (S2), tending towards 1.

**Table 2:** Comparative diversity analysis among the libraries SN1 and SN2 (using PAST version 1.77)

Diversity indices	SN1 (Chhiplakot)	SN2 (Munsyari)
Individuals	87	109
Dominance_D	0.3841	0.3894
Simpson_1-D	0.6159	0.6106
Shannon_H	1.176	1.239
Evenness_e^H/S	0.4629	0.4930
Menhinick	0.6705	0.7505
Margalef	1.279	1.344
Berger–Parker	0.4828	0.5505

**Fig. 4:**
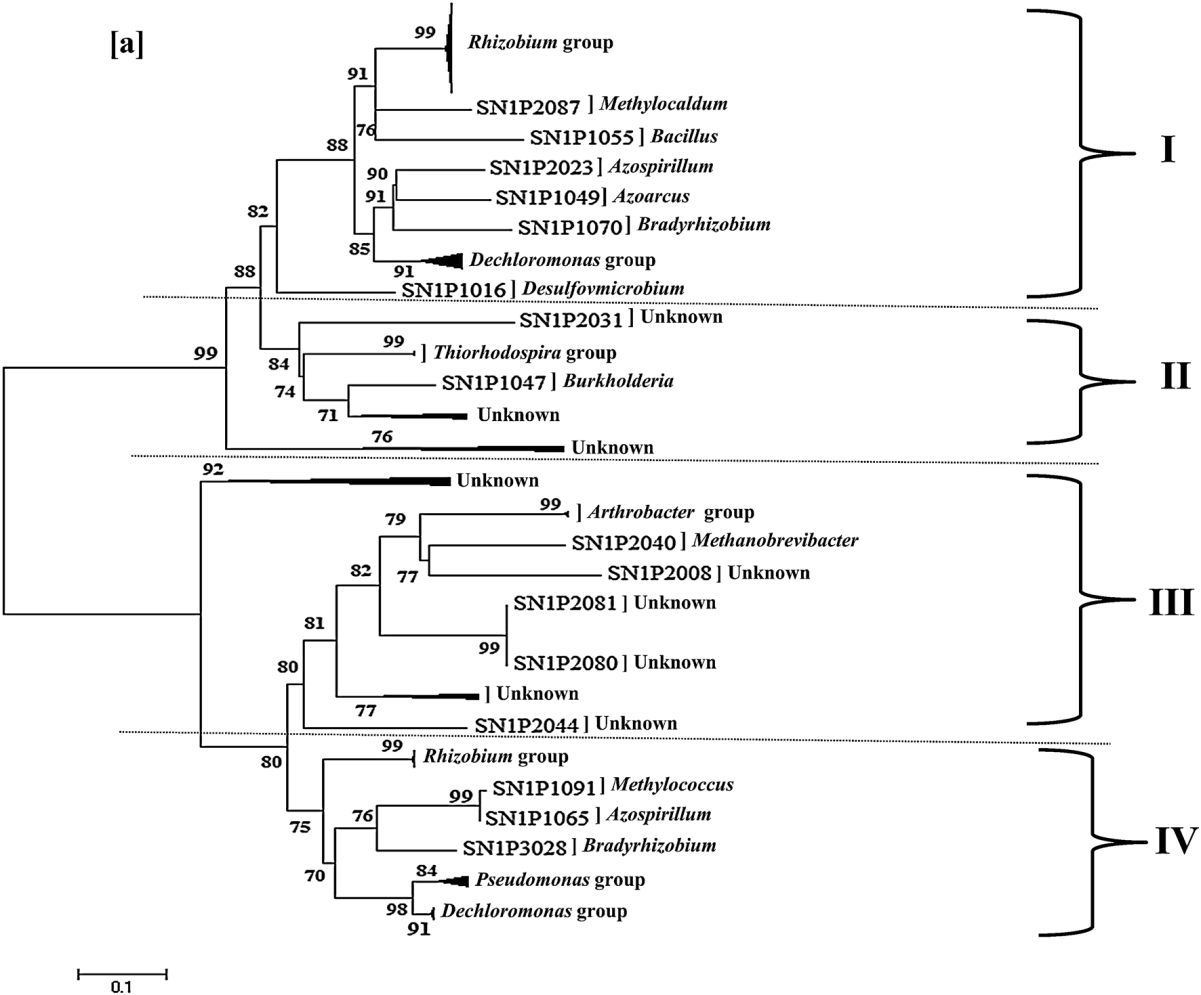
**a**, **b** Phylogenetic relationships among *nif*H sequences of SN1 and SN2 constructed from soil samples S1 and S2 by neighbor-joining method using MEGA 5 software package. The *scale bar* denotes 0.1 substitutions per site. The depths and widths of the wedges reflect the branching lengths and the numbers of clones within the clusters, respectively

The *nif*H amino acid sequences from SN1 and SN2 were compared with sequences in the databases and their phylogenetic relationships were investigated. Both the libraries SN1 and SN2 showed similar type of *nif*H distribution pattern. All the sequences were clustered into four major groups designated as groups I, II, III and IV (Fig. 4a, b). Group I was primarily composed by Proteobacteria sequences along with few Firmicute representatives. Group II consisted mainly of from Proteobacteria sequences, and *nif*H from unknown uncultivable sources; however, in case of SN2, cyanobacteria sequence was also clustered in it. Both the libraries differ significantly in terms of group III clustering. In case of SN1, it includes the sequences from Actinobacteria, archea and *nif*H from unknown origin; while that of SN2 possess *nif*H sequences from Actinobacteria, Deltaproteobacteria and cyanobacteria. Group IV of SN1 and SN2 was somewhat similar in terms of some common genera viz*. Rhizobium*, *Bradyrhizobium* and *Pseudomonas*; however, differs due to the presence of *Decholoromonas* cluster along with single sequence from *Methylococcus* and *Azospirillum* in SN1 only.

## Discussion

The Himalayas are considered as a reservoir of a diversified and dynamic gene pool. Additionally, the Himalayas may exert an “edge effect” as it lies along the ecotone of *Palearctic* and *Indo*-*Malayan* realms (Olson et al. 2001) and thus, among the hot spots of the world. Soil biological activity in these high-altitude agricultural lands is reportedly low due to suboptimal or freezing soil temperatures as seasonal freeze-thaw cycles changes various physicochemical properties of the soil providing both challenges and opportunities for the survival of indigenous microflora (Schmidt et al. 2009). However, the influence of climatic alterations on soil microorganisms has been rarely investigated, especially related to latitude (Yergeau et al. 2007). Margesin and Miteva (2011) reviewed the diversity and ecology of psychrophilic microorganisms and negatively correlate the bacterial abundance with the altitudes. It is important to note here that farmyard manure is the main source of agriculture fertilizer which helps replenish the soil fertility (Kumar et al. 2009). This provides an opportunity to native N_2_-fixing microorganisms for their survival and functionality (Coelho et al. 2008). The high copy number of *nif*H in the rhizospheric soils is in accordance with our previous study (Soni et al. 2010) and establishes good insight into correlation between nitrogen-fixing microorganisms and fertility of Himalayan agricultural soils of high altitude.

### Diazotrophic bacterial composition and diversity

Remarkably diverse *nif*H sequences were extracted from the RKB rhizosphere, and most of them were found to be novel and/or distantly related to those of cultivated organisms. The values of diversity indices pointed out the existence of more diverse diazotrophic assemblages in the RKB rhizosphere from Munsyari than that of Chhiplakot. It can be justified in terms of climate associated selection pressure which is more prominent at high altitudes hypothesizing that the abiotic stresses could be stronger determinants of microbiota composition than plants (Philippot et al. 2013). High abundance of Alphaproteobacteria in both the soils S1 and S2 could be explained by the fact that rhizosphere of RKB plants are frequently inhabited by different rhizobial symbionts (Valverde et al. 2006; Diaz-Alcántara et al. 2013). Proteobacteria especially *Rhizobium* and *Dechloromonas*, which comprise the majority of the cloned sequences, indicated their dominance in Himalayan rhizospheric soils.

Previous culture-dependent studies confirmed that WIH soil has bacterial communities with a tremendous potential of biodegradation (Soni et al. 2008; Goel et al. 2008) and plant growth promotion (Pandey et al. 2006; Selvakumar et al. 2011). These rare bacterial communities and their habitats are being threatened directly and indirectly as a result of global warming, increasing human intervention and especially new farming practices as revealed by the values of the Simpson index. Therefore, there is an immense need to explore and preserve the microbial diversity from these high-altitude agro-ecosystems.

The *nif*H phylogeny was found to be similar to those from previous recognized studies with respect to the *nif*H clustering pattern (Zehr et al. 2003). However, making of subclusters as per these earlier studies was not possible because of the lower number of sequences and absence of important marker genera in clone libraries viz. *Azotobacter* for *vnf*H. Both the clone libraries showed an early saturation indicating that there were no more OTUs available in the libraries which could be correlated with the values of Simpson index which revealed the tendency of homogeneity and, therefore, the saturation risk in the Himalayan agro-ecosystems.

Analysis of clone libraries revealed the dispersion of Alphaproteobacteria (*Rhizobium*, *Azospirillum*, *Bradyrhizobium) nif*H sequences into two different groups: group I and IV. Similarly, in SN1 genus *Dechloromonas* was also clustered into two groups I and IV. Moreover, cyanobacteria *nif*H sequences were found to be distributed in two major groups II and III of SN2. Additionally, the sequences of *Bacillus* (Firmicutes) were found clustering with sequences of *Methylocaldum* (Gammaproteobacteria) as well as *Rhizobium* (Alphaproteobacteria) in Group I of SN1. Genus *Arthrobacter* (Actinobacteria) was found to cluster with *Methanobrevibacter* (archea) in SN1 and cyanobacteria group in SN2. At the same time, though belong to same phylogenetic group (16SrRNA based), *nif*H sequences of *Rhizobium* and *Bradyrhizobium* were separated apart in both the libraries. These anomalies between *nif*H phylogeny and ribosomal RNA phylogeny could be the result of lateral gene transfer among microorganisms. It is not clear whether these nitrogenase genes from one cluster can be distinguished from that of other. But, this type of distribution of single genera in multiple clusters may be justified in terms of the presence of pseudo-*nif*H and/or *nif*H like gene(s) that are considered to function in some process other than nitrogen fixation (Zehr et al. 2003; Soni and Goel 2011). These homologs are either extraordinarily conserved in evolution or have been exchanged between the organisms relatively recently in evolutionary time (Soni and Goel 2011). In addition to this, many microorganisms may have multiple copies of nitrogenase genes, *nif* homologues and alternative nitrogenase (Zehr et al. 2003) which finally lead to the scattering of related groups within *nif*H based phylogenetic trees. Zehr et al. (2003) reviewed the broad patterns of N_2_-fixing phyla across multiple environments and reported the similar pattern of distribution of genera among the clusters viz. occurrence of single genera in multiple groups, clustering of cyanobacteria, Firmicutes and archaea with Proteobacteria members etc. However, for the first time, cyanobacteria were found to affiliate with more than one group. This novel clustering pattern of cyanobacteria *nif*H sequences will definitely enrich the *nif* database; thus, proves useful for future explorations.

## Conclusion

In conclusion, this study provides the qualitative as well as quantitative assessment of RKB associated N_2_-fixing bacterial diversity and identifies the two diazotrophic hotspots namely “Chhiplakot and Munsyari” from WIH. The isolation and characterization of indigenous populations of rhizobia from these regions may lead to the selection of inoculants strains for Himalayan high-altitude agricultural systems as substantiated by strain S10724. The study also holds promise in documenting the Himalayan agriculturally important microbial wealth that can be helpful for determining the diaztrophic community structure from the Himalayan RKB rhizosphere. In addition to this, these findings will definitely enrich our understanding about *nif*H taxonomy, classification and thus evolution.

## Electronic supplementary material

Below is the link to the electronic supplementary material.

Supplementary material 1 (DOCX 29 kb)
