# Supplementary material for: Diversified diazotrophs associated with the rhizosphere of Western Indian Himalayan native red kidney beans (Phaseolus vulgaris L.)
Source: 3 Biotech. 2014 Jul 24;5(4):433–41. doi: 10.1007/s13205-014-0238-5 (PMC4522724; doi:10.1007/s13205-014-0238-5)
Supplement: Supplementary file 1 — Supplementary material 1 (DOCX 29 kb) [file 13205_2014_238_MOESM1_ESM.docx]

**Table SM1** comparative *nif*H abundance in different soil samples used in this study as revealed by qPCR analysis. Each value is the mean of three replicates. Values in parentheses indicate standard error

| **Sr.**  **No.** | **Sample site** | **Latitude, longitude** | **Elevation**  **(meters)** | **Climate** | **Copy No. of *nif*H gene**  **(per g of soil)** |
| --- | --- | --- | --- | --- | --- |
| **1** | **Chhiplakot** | **30.06°N, 79.01°E** | **3090** | **Temperate** | **1.07 x 10^7^ (± 1.2 x 10^3^)** |
| **2** | Kala Muni Hilltop | 30.45°N, 80.30°E | 2700 | Temperate | 3.04 x 10^6^ (± 1.12 x 10^3^) |
| **3** | Mukteshwar | 29.28°N, 79.39°E | 2311 | Temperate | 1.29x 10^5^ (± 2.18 x 10^3^) |
| **4** | **Munsyari** | **30.07°N, 80.23°E** | **2200** | **Temperate** | **5.28 x 10^6^ (± 2.98 x 10^3^)** |
| **5** | Tejam | 30.52°N, 80.26°E | 2135 | Temperate | 4.3 x 10^6^ (± 1.68 x 10^3^) |
| **6** | Chaukori | 29.50°N, 80.20°E | 2010 | Temperate | 7.8 x 10^4^ (± 4.68 x 10^3^) |
| **7** | Vridh Jageshwar | 29.32°N, 79.24°E | 2000 | Temperate | 1.99x 10^5^(± 3.21 x 10^3^) |
| **8** | Kanda | 29.77°N, 79.87°E | 1900 | Temperate | 2.57 x 10^3^ (± 3.79 x 10^3^) |
| **9** | Paton Village | 29.35°N, 80.11°E | 1900 | Temperate | 2.42 x 10^5^ (± 1.63 x 10^3^) |
| **10** | Kausani | 29.80°N, 79.60°E | 1890 | Temperate | 2.28 x 10^4^ (± 0.98 x 10^3^) |
| **11** | Naini Village | 29.30°N, 79.25°E | 1800 | Sub-Temperate | 1.04 x 10^4^ (± 4.68 x 10^3^) |
| **12** | Thal | 29.81°N, 80.13°E | 1772 | Sub-Temperate | 3.32 x 10^5^ (± 1.55 x 10^3^) |
| **13** | Berinag | 29.80°N, 80.00°E | 1740 | Sub-Temperate | 1.27 x 10^5^ (± 3.76 x 10^3^) |
| **14** | Didihat | 29.49°N, 80.18°E | 1725 | Sub-Temperate | 4.09 x 10^5^ (± 1.51 x 10^3^) |
| **15** | Nachni | 29.98°N, 80.14°E | 1700 | Sub-Temperate | 4.17 x 10^4^ (± 2.55 x 10^3^) |
| **16** | SheetlaKhet | 29.36°N, 79.30°E | 1700 | Sub-Temperate | 2.68 x 10^3^ (± 1.17 x 10^3^) |
| **17** | Majkhali | 29.59°N, 78.96°E | 1670 | Sub-Temperate | 8.57 x 10^4^ (± 2.11 x 10^3^) |
| **18** | Madkot | 30.50°N, 80.10°E | 1650 | Sub-Temperate | 1.25 x 10^5^ (± 2.43 x 10^3^) |
| **19** | Askot | 29.76°N, 80.35°E | 1616 | Sub-Temperate | 4.3 x 10^5^ (± 2.08 x 10^3^) |
| **20** | Dharchula | 29.85°N, 80.50°E | 1615 | Sub-Temperate | 7.9 x 10^5^ (± 2.72x 10^3^) |
